# Non-Linear Probabilistic Modification of Miner’s Rule for Damage Accumulation

**DOI:** 10.3390/ma14237335

**Published:** 2021-11-30

**Authors:** Łukasz Blacha

**Affiliations:** Department of Mechanics and Machine Design, Opole University of Technology, 45-271 Opole, Poland; l.blacha@po.edu.pl; Tel.: +48-77-449-8075

**Keywords:** fatigue, damage accumulation, failure probability

## Abstract

A non-linear modification to Miner’s rule for damage accumulation is proposed to reduce the scatter between experimental fatigue life and fatigue life predicted according to the original Miner’s sum. Based on P-s-n probability distribution and design s-n curves, the modification satisfies the assumption of equality between the mean damage degree (at the critical level) and fatigue life random variables, which is not covered in the original formulation. The adopted formulation shows the discrepancies between the fatigue lives predicted according to the design s-n curves and the estimated probability distribution. It also proves that it is inappropriate to apply a normal distribution to fatigue life analysis and that the model becomes non-linear only for non-normal distributions. The predictions according to the established model were compared to the predictions obtained with Miner’s rule.

## 1. Introduction

The estimation of the degree of accumulated fatigue damage (damage degree) is necessary for the most common deterministic approach for the fatigue life analysis of structural materials under variable amplitude loading [1,2]. In general, the existing damage accumulation rules are based on models that can be classified into two categories—micro-scale and phenomenological [1,2]. The modeling of microscale mechanisms is still a challenging issue due to their complexity arising from the diversity of fatigue crack mechanisms and the randomness of material microstructures. The data needed for the calculation process may not be fully available in standard application situations, which, together with their complexity, makes these models difficult to apply in practical engineering. As an alternative, from the phenomenological perspective, fatigue damage is treated as a time- and loading-dependent process. It is assumed that damage gradually accumulates along with the loading cycles and that a developing crack will result in failure when the accumulated damage exceeds its critical value. Estimates of the accumulated damage are obtained mostly on the basis of the widely available stress–life (s-n) curves. The fact that these curves have been identified by the least squares method [3] leads to the inappropriate assumptions that 50% of the experimental lives are on the left side of the reference s-n curve, and 50% of the experimental lives are on the right side and that the normal distribution is appropriate for modeling fatigue damage. This issue could be overcome by, for example, implying a probabilistic stress–life curve, as was done in Reference [4], or a non-linear s-n curve, as was done in Reference [5], with the median interpolated according to the Weibull distribution identified by the maximum likelihood estimation. However, the latter can be viewed as being too complex for engineering approaches, mostly due to the problematic identification of the likelihood function. Nevertheless, in order to maintain the simplicity of the widely used ASTM standard [3], the modified approach should correspond to the existing s-n curves.

While analyzing the failure probability–stress–number of load cycles (P-s-n) non-normal probability distributions, it can be concluded that the number of cycles corresponding to the mean damage degree does not correspond to the mean fatigue life. At the same time, significant scatter of the computed damage degree versus the mean life can be observed. Although considerable studies have been performed on statistical models of fatigue damage accumulation, they often can be viewed as having a high level of complexity (e.g., References [6,7,8]).

The aim of the present study was to propose a simple way to modify the stress-related damage accumulation rules in order to better reflect the nature of the variance in experimental fatigue life and, as a result, to decrease the scatter between the predicted and experimental life. The approach is based on the probability distribution for a one-stage loading.

## 2. Relation between Fatigue Damage Accumulation and Fatigue Life

The fatigue life depends on the amount of damage the material can accumulate. Since this measurement cannot be physically obtained, an appropriate deterministic measure is usually introduced. Logically, the damage measure should take into account the applied load (or its result) and the loading duration. The most common measure is the damage degree, *d*. In the historically first, phenomenological Miner’s rule, the degree of damage accumulated during one loading stage, can be obtained according to the following equation:

(1) $$d=n/n_{f},$$

where:

*n*—the number of cycles in the loading stage;

*n_f_*—the s-n curve number of cycles to failure.

In case of a multi-stage loading, it is rewritten as follows:

(2) $$d=\sum_{i=1}^{j}n_{i}/n_{fi},$$

where:

*i*—the index of the loading stage;

*j*—the number of loading stages.

**Figure 1:**
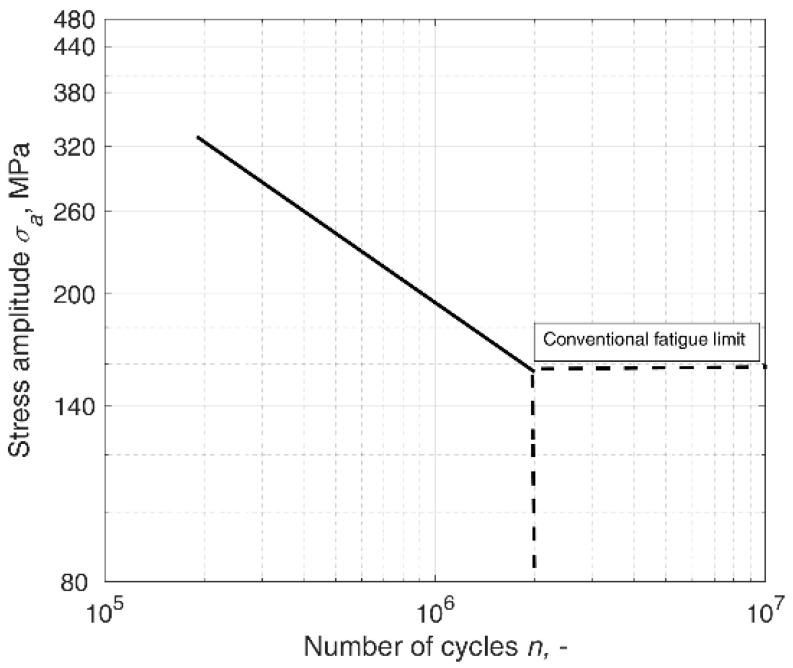
An example of an s-n curve.

Here, the information on the applied load is hidden in the *n_f_* value, whereas the duration of the applied load is given in the *n* value. The value of the number of cycles to failure *n_f_* is derived according to the s-n curve, relating the applied constant amplitude load to the fatigue life (Figure 1). Most frequently, in such formulations, it is assumed that the fatigue failure occurs when the damage degree reaches the critical value of *d* = *d_c_* = 1, which applies also to Miner’s rule.

As can be seen, Miner’s rule is based on a linear model of damage accumulation. Although Miner’s rule is widely used in the case of metallic materials because it is easy to implement and computationally inexpensive, an issue was raised concerning the scatter of the critical damage degree [1]. Linear damage accumulation rules, under certain circumstances (e.g., sequential loading effects, notches, non-standard environments), can produce significant and unusual scatter, corresponding to the scatter band equal to three or even more [9,10]. Non-linear damage accumulation rules offer more accurate or more conservative predictions than the linear rules [9,10]. They also seem to be physically justified since the fatigue processes involve crack nucleation, crack propagation, and final failure.

In 1956, Corten and Dolan formulated a power–law relationship for damage accumulation, which allowed for accounting for load effects [11]. Corten–Dolan’s model is also widely used and, being stress level-dependent, it is considered to be more universal. Nevertheless, the determination of its parameters can be troublesome, as they correspond to microcosmic damage theory and damage propagation. Later, the model was also adopted and developed by other researchers, including Marsh and Mackinnon [12], Chen [13], and, more recently, by Zhu et al. [14]. Their attempts have focused mostly on the re-definition of the exponent interpreted as the inverse slope of a hypothetical s-n curve. Zhu et al. [14] have introduced a dynamic Corten–Dolan’s model and demonstrated the better consistency of their model, although the determination of the introduced parameters for load interactions can be troublesome (approximates have been given). Furthermore, the definition of the introduced initial strength parameter seems to lack physical justification.

The s-n curve is also being used in models based on isodamage lines (e.g., by Subramanyan in Reference [15]). This group of approaches seems to be consistent and easy to interpret; however, in the underlying assumptions, it can be found that the s-n curve corresponds to a failure probability equal to 1.

Constant research is also being conducted on the group of damage measures other than the damage degree, for example, evaluations on the plane of maximum normal or shear strains, such as the Smith–Watson–Topper (e.g., Reference [16]) and Fatemi–Socie [17] parameters. Both of them utilize the Coffin–Manson equation as the fatigue curve. Difficulties of the practical application of these approaches arise mostly because the algorithm for finding the critical plane orientation is complicated. It also must be noted that not all of the damage can be evaluated on the critical plane [18].

Apart from those previously mentioned, non-standard approaches are also being developed (e.g., thermodynamic-based theories [19]), but they are often difficult in practical applications due to the use of non-standard fatigue curves or the introduction of additional material constants (e.g., Reference [20]).

From a statistical point of view, the reason for the scatter behavior of the critical damage degree lies in, among others, not considering the realistic fatigue life distribution. Another reason lies in the fact that the present formulation does not differentiate the damage degree between the stress levels, as well as it disregards possible material hardening at the first loading stages. It may not matter if the load spectra have similar character, but it can influence the results when the spectra are more diverse [21,22]; however, these effects do not arise in all materials [23].

## 3. Probability-Modified Fatigue Damage Accumulation Model

The linear formulation of Miner’s rule narrows down the possible solutions for a problem. As an alternative, assume that the damage accumulation rule in the life region before the knee point follows a power–law formulation, which makes it capable of accurately modeling the damage accumulation process in the wide range of loading cycles [9]:

(3) $$d=b\;\cdot \;n^{a},$$

where:

*a*, *b*—parameters corresponding to the loading.

In case of a multi-stage loading, it is rewritten as follows:

(4) $$d=\sum_{i=1}^{j}b_{i}\;\cdot \;n_{i}{}^{a_{i}},$$

where:

*a_i_*, *b_i_*—values of the parameters for a given loading stage.

Suppose that the parameter *b* can be defined as

(5) $$b=1/n_{f}.$$

From the probabilistic point of view, the critical damage degree should be viewed as the mean of the damage degree probability distribution, $\bar{d}=1$, and it should correspond to the mean fatigue life, $\bar{n}$. Due to the application of the least squares approach in the s-n curve generation process, the mean fatigue life is equal to the s-n curve number of cycles to failure only when the mean life in the assumed distribution of the fatigue life is the same as the median life. This is because the method of least squares is an approach in regression analysis that assumes the normal distribution of a set of experimental points. In the case of symmetrical probability distributions, such as a normal distribution, the mean life and the median life are the same. Normal, as well as log-normal or Weibull, distributions are usually assumed to be the distributions appropriate for estimating the probability of failure and the mean life. Due to the reasons already stated, the application of a normal distribution remains unjustified, which can be seen in Equation (4). Here, the exponent *a* would be equal to 1 due to the equality between the mean and the median life, which automatically implies Miner’s rule with all of its inconveniences. In this way, the critical damage degree corresponds to the number of cycles from the reference s-n curve. In the proposed model, it is assumed that the critical damage degree remains at the same level (i.e., *d_c_* = 1), but the corresponding number of load cycles is shifted to lower values that result from the probabilistic s-n curve. The probabilistic s-n curve has its source in the appropriate probability distribution for the fatigue life. From the studies performed on P-s-n distributions (e.g., References [24,25,26,27]), it can be found that, in most cases, researchers have applied different variants of a Weibull distribution. A variant of a Weibull distribution tailored for a logarithmically distributed random variable of the fatigue life of steel welded joints in a one-stage loading (i.e., a constant amplitude) was proposed by the author of Reference [4]. In general, the probability of failure *P_f_* in this variant can be described in the following manner [28]:

(6) $$P_{f}=1-exp\left(-{\left(\log\left(n\right)/h_{l}\right)}^{g_{l}}\right),$$

where:

*h_l_* = log(*n_f_*)—scale parameter, s-n curve fatigue life;

*g_l_* = *p*/log(*n_f_*)—shape parameter;

*p*—parameter of the distribution estimation, constant within the entire s-n curve.

**Figure 2:**
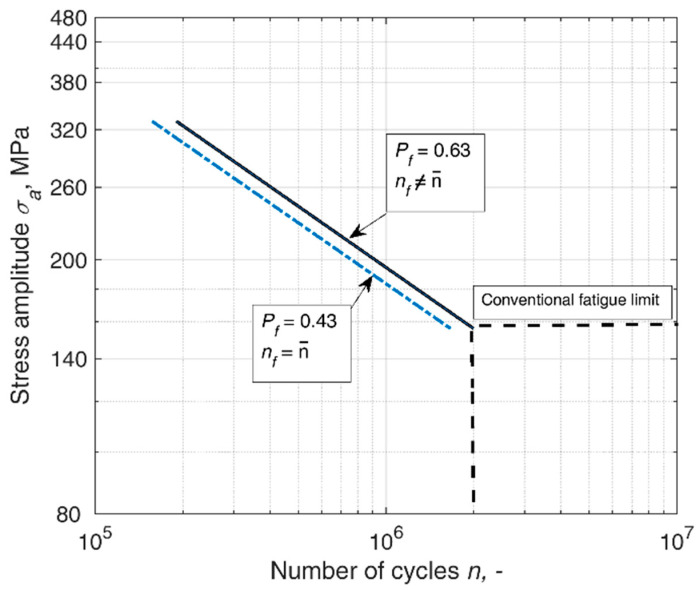
An example s-n curve of fatigue lives and the corresponding probability of failure in the Equation (6) Weibull formulation of life distribution. Mean fatigue lives correspond to the s-n curve shifted to *P_f_* = 0.43.

In Equation (6), fatigue lives lying on the s-n curve do not correspond to the 50% probability of failure (i.e., the median life), which can be seen in Figure 2 and compared to Figure 1.

In order to match the mean damage degree $\bar{d}=1$ with the mean fatigue life $\bar{n}$, Equation (3) should be rewritten in a logarithmic space, as follows:

(7) $$d_{l}=\log\left(b\right)+a\;\cdot \;n_{l},$$

where:

*d_l_*—logarithm of *d*, *d_l_* = log(*d*)

*n_l_*—logarithm of *n*, *n_l_* = log(*n*).

It is worth mentioning that Equation (7) can also serve as a linear transfer function between the logarithm distributions of the fatigue life and the damage measure random variable.

**Figure 3:**
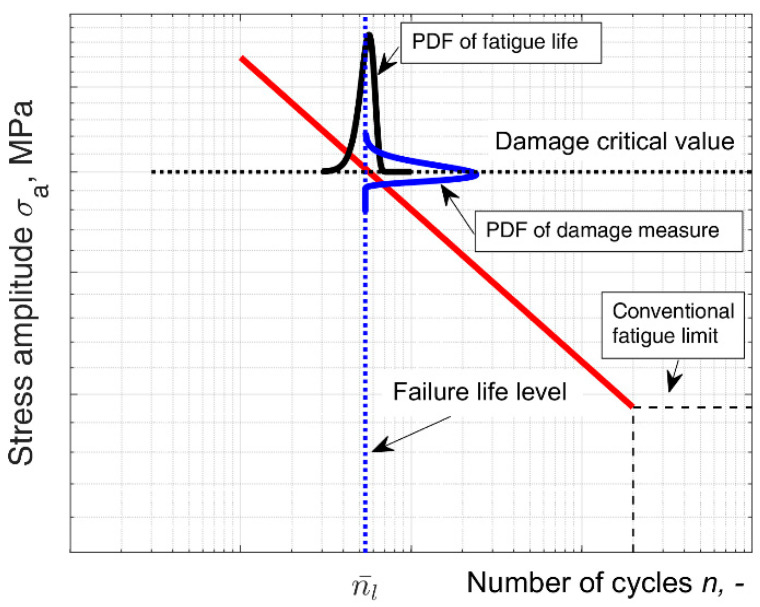
Correlation between probability density functions (PDFs) for fatigue life and damage degree probability distributions.

Due to the fact that the process of fatigue degradation of the material is non-stationary [29,30,31], the number of load cycles that represent the mean fatigue life corresponds to the mean damage degree only when it accumulates to its failure threshold—the damage degree critical value. Thus, the mean fatigue life directly corresponds to the mean damage degree only at the level of the damage critical value (Figure 3):

(8) $$\bar{d_{l,c}}=\log\left(b\right)+a\;\cdot \;\bar{n_{l}},$$

where:

$\bar{d_{l,c}}$—the mean damage degree critical value, $\bar{d_{l,c}}=\log\left(1\right)$ in a one-stage loading.

Based on the above, a relation for the exponent *a* in the proposed damage accumulation rule (Equation (3)) can be formulated as follows:

(9) $$a=-\log\left(b\right)/\bar{n_{l}}.$$

It should be noted that, because the relation for *b* is a function of stress (Equation (5)), such a formulation determines parameter *a* as stress-dependent. From Equation (9), it can be seen that the exact value of *a* also depends on the distribution of the fatigue life. While to some extent, the above approach resembles the one by Sun et al. [32], the considerable differences can be found in the following underlying assumptions: (i) here, the power–law exponent *a* is precisely defined and refers only to the number of cycles at the given loading stage; (ii) due to the above, the damage degree critical value is assumed to be constant on a given stress level, which makes the calculations less time consuming.

Compared to the existing models listed in Section 2, the proposed model has three advantages—(i) the suggested non-linear formulation is based on the transformed linear transfer function for the probability distribution of a logarithm random variable rather than subjective experiences, so it has rigorous derivation, a theoretical basis, and an additional field of application; (ii) the applied power–law exponent becomes a slope of the linear transfer function, which makes it constant at a given stress level and constitutes this rule as capable of quantifying the dispersion of fatigue damage, which is dynamic during the process of fatigue damage accumulation; and (iii) it can be established based on the basic fatigue data of the material and the cumulative distribution function of the investigated stress level.

## 4. Model Validation

### 4.1. Methods

In order to demonstrate the advantages of the proposed modification, fatigue failure probability distribution, together with the uniaxial constant amplitude loading fatigue test data, was adopted from Karolczuk and Palin-Luc [28], Gao and Yuan [33], and Xie et al. [34]. Karolczuk and Palin-Luc investigated the fatigue lives of 1.0570 steel, which allowed them to estimate the P-s-n Weibull distribution for log fatigue lives (i.e., $\log\left(N\right)\sim W\left(h_{l},g_{l}\right)$); Gao and Yuan re-published the data for the P-n Weibull distribution for the fatigue lives of aluminium alloy LY12-CZ (i.e., N~W(h,g)), which was originally published by Ji and Yao [35], while Xie et al. demonstrated the normal P-s-n distribution for the log fatigue lives (i.e., log(N)~N(μ,σ)) of aluminium alloy 2524-T3; in their paper, the distributions were abbreviated as D1, D2, and D3, respectively.

The above data was used to generate cumulative distribution functions at different stress levels, as well as to investigate the differences between Miner’s damage degree and the modified damage degree. The cumulative distribution functions were analyzed in order to:

- determine whether the probability of failure corresponding to the same damage degree changes along with the change in stress level, that is, whether the proposed modification is stress level-dependent;
- compare the probabilities of failure: corresponding to the mean damage degree critical value to the one corresponding to the mean fatigue life, in order to conclude whether they are equivalent or not; and
- determine the relationship between the damage degree and the failure probability.

Due to the limited applicability of the P-n distribution (D2), validation involved different stress amplitudes, $\sigma_{a}=\left\{200;\;300;\;350\right\}\mathrm{MPa}$ (distributions D1 and D3) and $\sigma_{a}=\left\{113;\;167;\;196\right\}\mathrm{MPa}$ (distribution D2). Using the above data, the damage degree d was calculated according to the following s-n curve model:

(10) $$n_{f}={\left(C/\sigma_{a}\right)}^{m},$$

where:

$\sigma_{a}$—stress amplitude;

*C*, *m*—material parameters.

The material parameters concerning 1.0570 steel can be found in Reference [28], whereas, in the case of the LY12-CZ and 2524-T3 materials, the s-n curve was derived by the least squares polynomial curve fitting, according to the published test data [33,34,35]. The corresponding values are shown in Table 1.

The estimated distribution parameters concerning distribution (D1) and the corresponding test data are widely available and can be found in Reference [28]. The log-Weibull probability distribution used herein is defined through the scale parameter h = log(*n_f_*) (Equation (6)), determined by the s-n curve in Table 1, and the shape parameter g = p/log(n_f_), with a parameter *p* value of 580, which was estimated as the best correlation between the experimental and calculated fatigue lives. This fit allowed for the lives for *P_f_* = 0.63 to agree with the s-n curve fatigue lives (*P_f_* = 1 − exp(−1) = 0.63) and for the scatter band defined by *P_f_* = {0.05; 0.95} to agree well with the experimental scatter band [28]. The scale and shape parameters estimated for the considered stress levels can be found in Table 2.

In distribution (D2), failure probability *P_f_* is obtained according to the Weibull cumulative distribution function, as follows:

(11) $$P_{f}=1-exp\left(-{\left(n/h\right)}^{g}\right),$$

where:

*h*—scale parameter,

*g*—shape parameter.

The parameter estimation results are shown in Table 3 [34].

The log-normal distribution was used to obtain the failure probability *P_f_* for the third material being analyzed (distribution D3):

(12) $$P_{f}=\frac{1}{2}+\frac{1}{2}erf\left(\left(\ln\left(n\right)-\mu \right)/\surd 2\sigma \right),$$

where:

*erf*—Gauss error function;

*μ*—mean value; here, $\mu =\bar{n}$ [35].

The estimated parameters can be found in Table 4. Mean log life $\bar{n_{l}}$ was calculated as a common logarithm of the mean $\bar{n}$ in Reference [35].

The model validation process requires knowledge of the amount of accumulated damage. The parameter estimation results listed in Table 1, Table 2 and Table 3 were used to establish the model characterizing fatigue damage accumulation, which was obtained according to Equation (3). The underlying principle of the mean value equivalence (Equation (8)) assumes knowledge of the power–law exponent *a*, which can be quantified by the mean value of fatigue life. In the condition of a given stress level, the mean value of fatigue life in the linear space can be predicted with the knowledge of the relation for failure probability, as follows:

(13) $$\bar{n}=\int_{0}^{\infty }\left(1-P_{f}\left(n\right)\right)dn.$$

In the logarithmic space, it can be predicted by the following:

(14) $$\bar{n_{l}}=\int_{-\infty }^{\infty }\left(1-P_{f}\left(n_{l}\right)\right)dn_{l}.$$

The mean values of fatigue life under the investigated stress levels were determined according to the above equations and are summarized in Table 5. The value ascribed to distribution (D2) was calculated as a common logarithm of the obtained mean $\bar{n}$.

**Figure 4:**
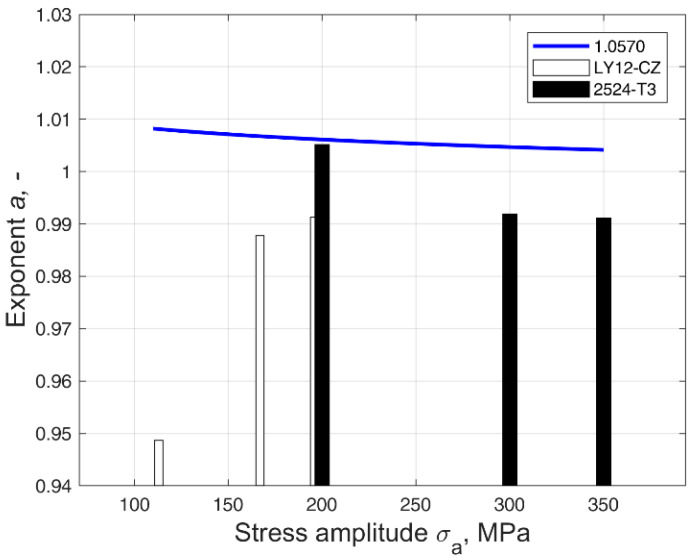
Value of the exponent *a* in the cases of the 1.0570 steel and the LY12-CZ and 2524-T3 aluminium alloys in relation to the stress amplitude.

The derived mean values, together with the corresponding s-n curves, were then used to determine the values of the parameter *b* and exponent *a*, according to Equations (5) and (9), respectively. A plot of the values of exponent *a* changing along the stress amplitude can be seen in Figure 4. Due to the limited availability of the data concerning the parameters of distributions (D2) and (D3) at different stress levels, the values were obtained only for the available data set and are presented in the form of vertical bars.

The investigation into the relationship between the damage degree and the failure probability involved only the one-stage loading, as this was assumed to provide more accurate insight into the relationship between the mean damage degree and the failure probability. As a result, the results are well-established, and issues related to the transition between different stress levels of constant amplitude cannot affect the results.

### 4.2. Results

**Figure 5:**
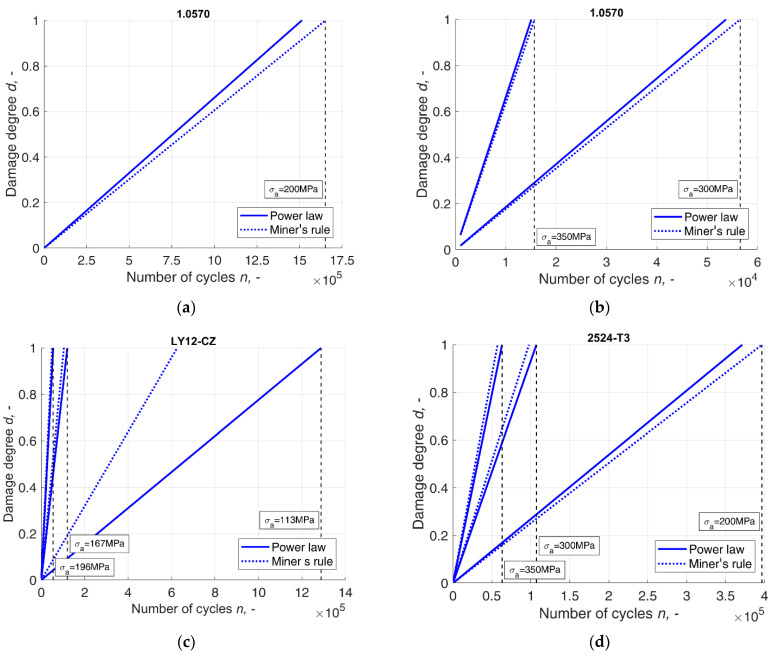
Differences between the Miner’s damage degree and the degree of damage calculated according to the proposed model, where (**a**,**b**) 1.0570 steel. Differences between the Miner’s damage degree and the degree of damage calculated according to the proposed model, where (**c**) LY12-CZ aluminium alloy; (**d**) 2524-T3 aluminium alloy.

The proposed probability-based model was applied to predictions of damage degree in different conditions. Here, the model was established for 1.0570 steel and two aluminium alloys, LY12-CZ and 2524-T3, according to Equation (3), the design s-n curves in Equation (10) and Table 1, and the parameter estimation results listed in Section 4.1, as well as Table 2, Table 3, Table 4 and Table 5. The calculated values of damage degree were further compared to the values calculated according to Miner’s rule. The comparison can be seen in a plot of the calculated values as a function of the number of cycles at the given stress levels, shown in Figure 5.

**Figure 6:**
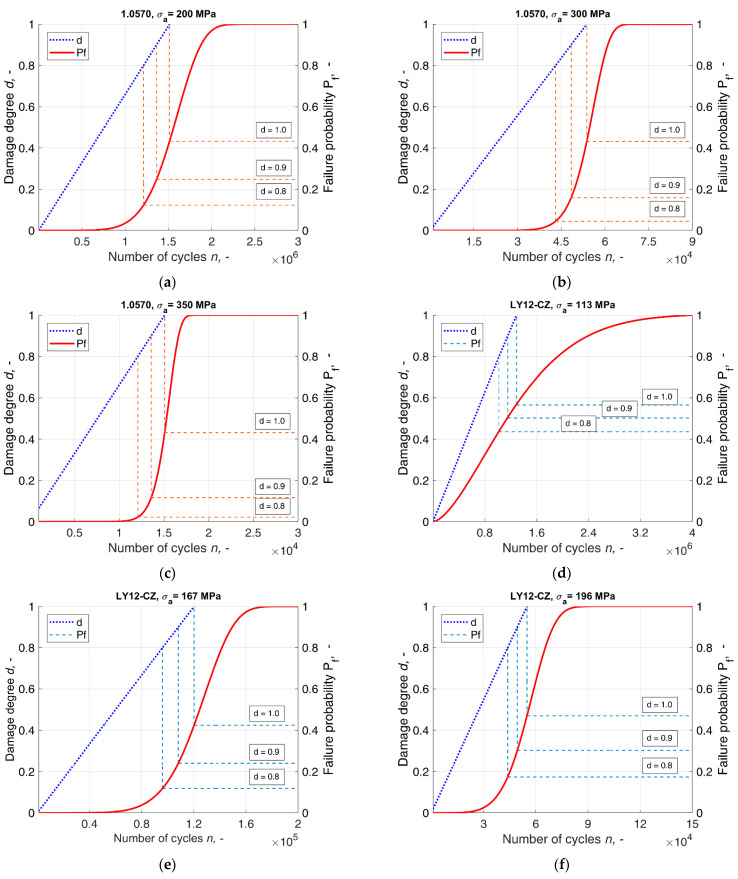

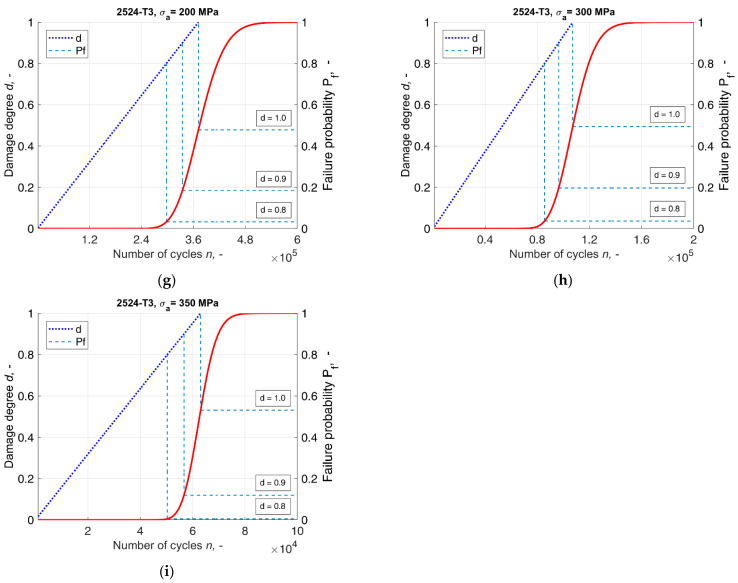
Comparisons between the probability of fatigue failure and damage degree at the different number of load cycles, where (**a**–**c**) 1.0570 steel. Comparisons between the probability of fatigue failure and damage degree at the different number of load cycles, where (**d**–**f**) LY12-CZ aluminium alloy. Comparisons between the probability of fatigue failure and damage degree at the different number of load cycles, where: (**g**–**i**) 2524-T3 aluminium alloy.

In order to verify the ability of the model to meet the aims of the validation process, a comparative analysis was performed between the degree of accumulated damage *d* and the fatigue failure probability *P_f_*. The analysis involved plotting the damage degree *d* in the domain of the load cycles at the investigated stress levels on a retained plot of the corresponding cumulative distribution function. A view of the above functions obtained during the validation process can be found in Figure 6. The process allowed for an investigation into the possible correlation between the damage degree and the failure probability. The probabilities derived at three levels of damage degree *d* = {0.8; 0.9; 1.0} are listed in Table 6.

## 5. Discussion

In this study, a non-linear modification of the well-known Miner’s rule was proposed with the aim of reducing the scatter of the damage degree and investigating the relationship between the accumulated damage and the probability of failure. The proposed solution demonstrated superiority over Miner’s rule in two areas—determining the relationship between the failure probability and the damage degree and illustrating the tendency of the fatigue life random variable.

The results were obtained according to the underlying assumption of equivalence in the mean values of distributions of the fatigue life and the damage degree on the failure life level. As a result of this principle, the damage critical value corresponds to the mean fatigue life in any distribution, not only the standard normal distribution. In the case of this distribution, the model was reduced to the original Miner’s rule, which can serve as evidence for the inappropriateness of applying a normal distribution to fatigue analysis and relating the 0.5 probability to the fatigue life of the s-n curve. The mean value of the distributions used in the validation process in each case corresponded to probabilities other than 0.5, whereas, in the case of the distribution of a logarithm random variable, the results were found to have less scatter amongst the different stress levels. Comparing the probabilities that correspond to the damage degree values of 0.8 and 0.9 at different stress levels, it can be found that the probability differs amongst them, which validates the model as capable of reflecting the nature of the load–material interaction, which varies with different stress levels. In the case of the obtained damage degree, the biggest differences arose at the LCF region, as can be seen in Figure 5, which makes it consistent with the theory that damage in the LCF and HCF does not accrue at the same rate, and nor does it accrue linearly with the applied cycle fraction, as it was underlined by Halford [36]. This is the main advantage of the present model over the traditional Miner’s rule due to the fact that applying Miner’s rule usually provides unsatisfactory results with the history of loadings corresponding to the LCF region. In the case of such load histories, the predicted fatigue life is non-conservative [9,36], whereas the biggest differences between Miner’s rule and the proposed model were demonstrated to be in this region specifically. In the case of the $\log\left(N\right)\sim W\left(h_{l},g_{l}\right)$) random variable at the critical damage degree, the difference at the stress amplitude $\sigma_{a}=200\;$ MPa was about 1.5 × 10^5^ load cycles.

The introduced modified damage accumulation rule reflects the central tendency of the fatigue life random variable, which could help in reducing the scatter of the accumulated damage. At the same time, the approach maintains the simplicity of its traditional version, applying standard design s-n curves into the calculation process. Additional attention may be needed in more complex applications, such as random loading or high–low/low–high multi-stage loadings. The formulation of parameter *b* remains open and can be reformulated without additional re-definitions of the other parts of the model since the power–law exponent *a* is a function of not only the mean fatigue life, but it also depends on the value of this parameter.

The adopted form of the proposed relation for the damage degree could help in the future advancement of research undertaken in the field of the probability distribution of the damage degree for multi-stage loading. While the phenomena of damage accumulation cannot be directly quantified, the appropriate distribution should be transformed from the distribution of the fatigue life random variable. The approach requires the probability distribution to be of the same type during the entire loading history, which imposes linear transfer functions as the appropriate relation to adopt in order to transform the fatigue life distribution into the distribution of damage measure, such as the damage degree. At the same time, linear transfer functions impose linear damage accumulation rules, the application of which remains questionable, as was stated previously. A logarithmically distributed random variable may be the solution, which would allow for the linear transfer function to be maintained, while the applied damage accumulation rule is non-linear and described according to the proposed relation. The function proposed for quantifying the fatigue damage becomes linear when transformed into the log-space, which allows for an easy transformation of the distribution of logarithm random variables, for example, the log(*N*) random variable. Additional future research and fatigue tests are required in order to apply the developed damage accumulation rule and validate it as a probability distribution transfer function.

## 6. Conclusions

Based on the results obtained in one-stage loading conditions, the following conclusions can be drawn:

- The model evidenced the inappropriateness of applying a normal distribution to the fatigue life analysis of metallic materials, whereas non-linear growth of the accumulated damage was assumed in the cases of the other distributions.
- By applying the suggested modification, the mean critical damage degree at different values of stress amplitude in each case was found to correspond to the probability of failure other than the 0.50 probability in the original approach.
- The logarithmic definition of the fatigue life random variable demonstrated better consistency in the comparison results of the damage degree and failure probability.
- In each case of the material-loading combination, the values of damage degree *d* = [0.8; 0.9] at different values of stress amplitude correspond to different failure probabilities; thus, the modification was demonstrated to be capable of being stress level-dependent, in contrast to Miner’s rule.
- The proposed model reflects the central tendency of the fatigue life random variable, which may help to reduce the statistical scatter of the damage degree; at the same time, the approach retains the simplicity of Miner’s rule.
- The assumed relation for the damage degree may find the application as a transfer function, thereby enabling an estimation of the distribution of the damage degree random variable, followed by the distribution of the fatigue life random variable in multi-stage loading conditions.

## Figures and Tables

**Table 1:** Parameters of the s-n curve for the considered materials.

Parameters	Material		
	1.0570	LY12-CZ	2524-T3
*C*	1117.76	2069.69	8300.00
*M*	8.32	4.59	3.46

**Table 2:** Parameters of Weibull distribution for fatigue lives of 1.0570 steel.

Stress Amplitude $\sigma_{a}$, MPa	Parameters	
	Scale, *h_l_*	Shape, *g_l_*
200	6.2177	93.2824
300	4.7526	122.0384
350	4.1956	138.2399

**Table 3:** Parameters of Weibull distribution for fatigue lives of LY12-CZ aluminium alloy.

Stress Amplitude $\sigma_{a}$, MPa	Parameters	
	Scale, *h*	Shape, *g*
113	1,442,370	1.60
167	131,635	6.55
196	59,880.5	5.32

**Table 4:** Parameters of log-normal distribution for fatigue lives of 2524-T3 aluminium alloy.

Stress Amplitude $\sigma_{a}$, MPa	Parameters		
	Mean Life, $\bar{n}$	Mean Log Life, $\bar{n_{l}}$	Standard Deviation, σ
200	376,628	5.57	43,666
300	108,119	5.03	12,876
350	62,882	4.80	5353

**Table 5:** Log mean fatigue lives at the investigated stress levels.

Stress Amplitude $\sigma_{a}$, MPa	Material (Probability Distribution)		
	1.0570 (D1)	LY12-CZ (D2)	2524-T3 (D3)
113	-	6.11	-
167	-	5.08	-
196	-	4.74	-
200	6.18	-	5.57
300	4.73	-	5.03
350	4.18	-	4.80

**Table 6:** Correlation between the damage degree and probabilities of failure at different stress levels, where $\bar{P_{f}}$ is the probability of failure (arithmetic mean), $\Delta \bar{P_{f}}$ is the difference between the mean probability of failure and probabilities at the given stress level, and $\sigma_{a}$ is the stress amplitude.

**Damage Degree *d*, -**	Material (Probability Distribution)								
	1.0570 (D1)			LY12-CZ (D2)			2524-T3 (D3)		
	$\sigma_{a}$ **, MPa**	$\bar{P_{f}}$	$\Delta \bar{P_{f}}$	$\sigma_{a},\;\mathrm{MPa}$	$\bar{P_{f}}$	$\Delta \bar{P_{f}}$	$\sigma_{a},\;\mathrm{MPa}$	$\bar{P_{f}}$	$\Delta \bar{P_{f}}$
1.0	200		0	113		0.08	200		−0.02
	300	0.43	0	167	0.49	−0.06	300	0.50	−0.01
	350		0	196		−0.02	350		0.30
0.9	200		0.07	113		0.15	200		0.02
	300	0.17	−0.01	167	0.35	−0.11	300	0.17	0.03
	350		−0.06	196		−0.05	350		−0.05
0.8	200		0.06	113		0.19	200		0.01
	300	0.06	−0.02	167	0.24	−0.12	300	0.02	0.12
	350		−0.04	196		−0.07	350		−0.02

## Data Availability

The data presented in this study are available on request from the corresponding author.

## Abbreviations

*a*		power–law exponent,
*b*		loading parameter,
*C*		s-n curve material parameter,
*d*		degree of accumulated fatigue damage,
*d_c_*		critical value of accumulated fatigue damage,
*d_l_*		common logarithm for the degree of accumulated fatigue damage,
$\bar{d_{l,c}}$		critical value of mean damage degree (in log space),
*D*		degree of accumulated fatigue damage (random variable),
*g*, *g_l_*		shape parameter,
*h*, *h_l_*		scale parameter,
*n*		number of load cycles,
*n_f_*		number of cycles until fatigue failure,
$\bar{n}$		mean number of load cycles,
$\bar{n_{l}}$		mean number of load cycles (in log space),
*N*		number of load cycles (random variable),
*m*		s-n curve material parameter,
*σ*		standard deviation,
*σ_a_*		stress amplitude, MPa,
*P_f_*		failure probability.
